# Neural network-based processing and reconstruction of compromised biophotonic image data

**DOI:** 10.1038/s41377-024-01544-9

**Published:** 2024-09-04

**Authors:** Michael John Fanous, Paloma Casteleiro Costa, Çağatay Işıl, Luzhe Huang, Aydogan Ozcan

**Affiliations:** 1 Electrical and Computer Engineering Department, University of California, Los Angeles, CA USA; 2 Bioengineering Department, University of California, Los Angeles, CA USA; 3 California NanoSystems Institute (CNSI), University of California, Los Angeles, CA USA; 4 Department of Surgery, David Geffen School of Medicine, University of California, Los Angeles, CA USA

**Keywords:** Biophotonics, Microscopy

## Abstract

In recent years, the integration of deep learning techniques with biophotonic setups has opened new horizons in bioimaging. A compelling trend in this field involves deliberately compromising certain measurement metrics to engineer better bioimaging tools in terms of e.g., cost, speed, and form-factor, followed by compensating for the resulting defects through the utilization of deep learning models trained on a large amount of ideal, superior or alternative data. This strategic approach has found increasing popularity due to its potential to enhance various aspects of biophotonic imaging. One of the primary motivations for employing this strategy is the pursuit of higher temporal resolution or increased imaging speed, critical for capturing fine dynamic biological processes. Additionally, this approach offers the prospect of simplifying hardware requirements and complexities, thereby making advanced imaging standards more accessible in terms of cost and/or size. This article provides an in-depth review of the diverse measurement aspects that researchers intentionally impair in their biophotonic setups, including the point spread function (PSF), signal-to-noise ratio (SNR), sampling density, and pixel resolution. By deliberately compromising these metrics, researchers aim to not only recuperate them through the application of deep learning networks, but also bolster in return other crucial parameters, such as the field of view (FOV), depth of field (DOF), and space-bandwidth product (SBP). Throughout this article, we discuss various biophotonic methods that have successfully employed this strategic approach. These techniques span a wide range of applications and showcase the versatility and effectiveness of deep learning in the context of compromised biophotonic data. Finally, by offering our perspectives on the exciting future possibilities of this rapidly evolving concept, we hope to motivate our readers from various disciplines to explore novel ways of balancing hardware compromises with compensation via artificial intelligence (AI).

## Abstract (web summary)

This article reviews how deep learning compensates for compromised biophotonic measurement metrics, enhancing bioimaging in cost, speed, and form factor and improving parameters like field of view and depth of field.

## Graphical abstract

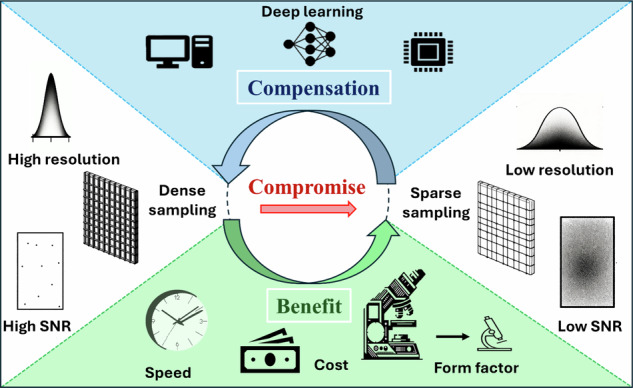

## Introduction

The integration of deep learning with biophotonic technologies^1,2^ heralds an unprecedented era in imaging and microscopy, characterized by transformative enhancements in the realm of image reconstruction^3,4^. Central to this innovative shift is the concept of neural network-based data processing, a powerful approach that has gained significant traction within the field of bioimaging. Neural network compensation hinges on a deliberate strategic compromise—a calculated choice to sacrifice certain measurement metrics in exchange for their later restoration or enhancement through the application of deep learning models trained on a substantial amount of data. This strategic trade-off can serve different purposes, including increasing temporal resolution and imaging speed, simplifying hardware configurations, and reducing costs. It also introduces novel ways to deal with typical peripheral bioimaging issues, such as phototoxicity^5^ and photobleaching^6,7^, a concern when dealing with sensitive biological specimens.

**Fig. 1:**
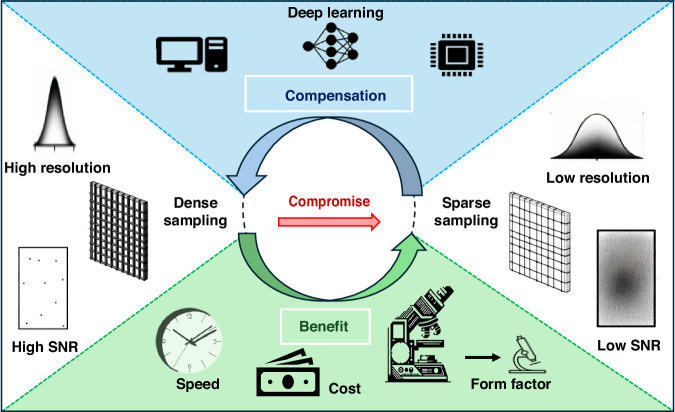
Schematic illustrating the concept of neural network-based image processing and reconstruction of compromised photonic data in terms of resolution, sampling density, and SNR. Deep learning compensation results in speed, cost, and/or size benefits

In this Review article, we cover a multitude of imaging systems^8–21^ that involve deliberate impairments, including to the point spread function (PSF), signal-to-noise ratio (SNR), sampling volume, and pixel resolution, which are recuperated along with enhancements to one or more of the following: spatial/temporal resolution, field of view (FOV), depth of field (DOF) and space-bandwidth product (SBP). These intricately calculated trade-offs, while necessitating the initial relinquishment of specific metrics, can offer significant practical benefits through the application of deep learning-based inference, as illustrated in Fig. 1.

Our exploration encompasses a review of over a dozen such biophotonic approaches, each of which skillfully leverages the compensatory capacities of deep learning^8–21^. These endeavors show how artificial intelligence (AI) can help overcome a variety of bioimaging challenges, furthering the field of biophotonics. Table 1 presents some of the main articles that are covered in this Review and highlights the *compromised/compensated* metrics involved in each case. Our extensive Review is an attempt to delineate the synergistic relationship that exists between deep learning and biophotonics and is divided into three sections: (i) *refocusing and deblurring*, (ii) *reconstruction with less data*, and (iii) *improving image quality and throughput*. Though there is some overlap among methods for each section, as indicated by the symbols listed in Table 1, we assigned each technique to the category most pertinent to the metrics that are conceded and subsequently restored or enhanced. Each segment comprises a few representative major studies that help illustrate the powerful assistance that deep learning can lend in advancing biophotonic technologies.

**Table 1:**
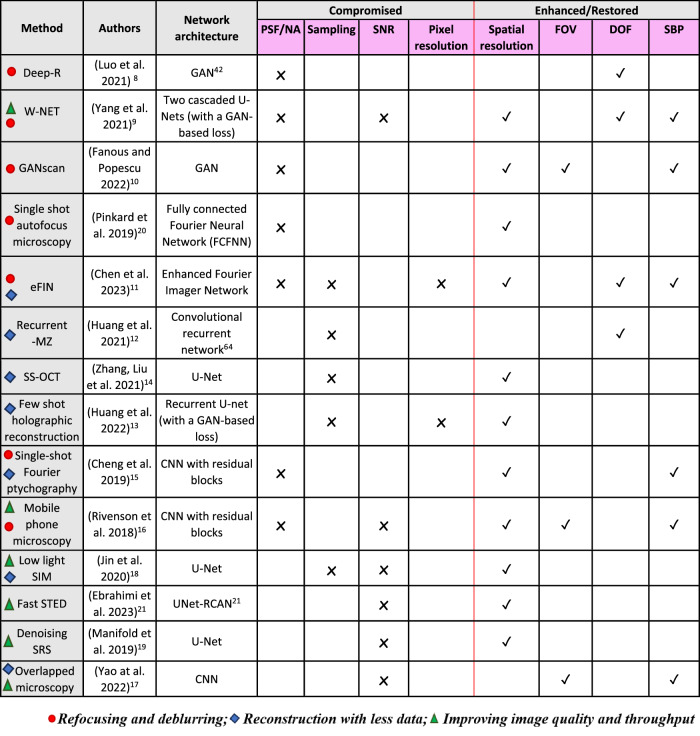
Neural network-based image processing and reconstruction methods using compromised data with the corresponding trade-offs and overall benefits^8–21,42,64^

## Refocusing and deblurring

**Fig. 2:**
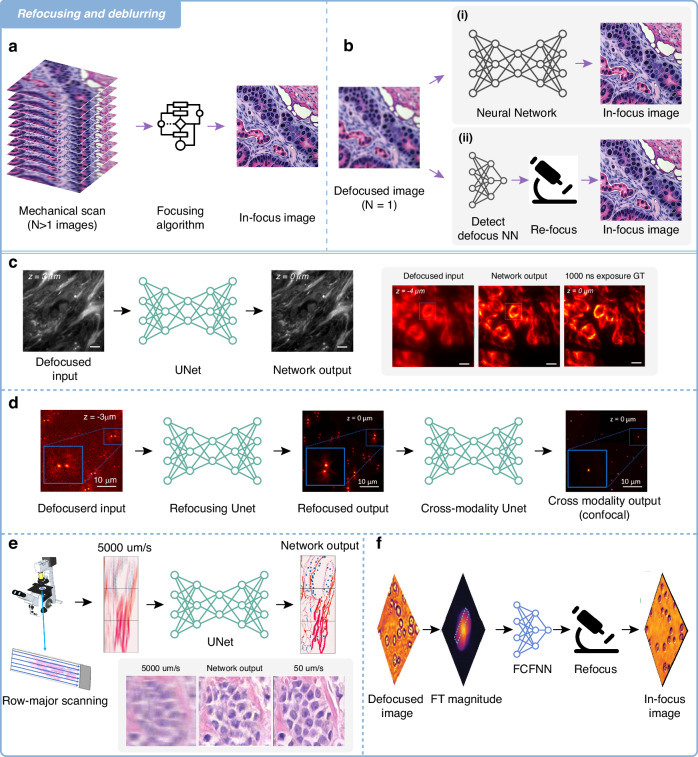
Refocusing and deblurring. **a** Traditional mechanical scanning technique where multiple defocused images are captured at different focal planes (*N* > 1 images). These images are then analyzed to identify the sharpest in-focus image, requiring extensive data acquisition and processing time**. b** Deep neural network-based approaches to image refocusing (computational or optical). **c** Deep-R blindly autofocuses a defocused image after its capture^8^. **d** Structure of W-Net, containing two cascaded neural networks: (1) virtual image refocusing network and (2) cross-modality image transformation network optimized for DH-PSF^9^. **e** The GANscan method resolves deliberately motion blurred scans that save on time using models trained with relatively slow scans^10^. **f** The FCFNN^20^ model uses just one coherent out-of-focus image, which is then analyzed through an established pipeline to acquire a focus prediction, following which the microscope’s optics are mechanically adjusted [figure adapted with permission from ref # 20 © Optical Society of America]

The process of obtaining high-fidelity, all-in-focus images without the artifact of motion-blur is crucial in the analysis of biophotonic data. Traditional refocusing methods, as illustrated in Fig. 2a, often rely on mechanical scanning techniques wherein multiple images are captured at different focal planes in a serial ‘stop-and-stare’ fashion. These images are then algorithmically analyzed to identify the best in-focus image and the focal position. This process requires extensive data acquisition and processing time.

There is also the method of *remote* focusing, which allows for rapid and precise adjustments to the focus without disturbing the sample^22,23^. This approach does not generally require physical movements of an entire piece of equipment, such as moving the objective lens or the sample mounting stage. Remote focusing also reduces the overall acquisition time and can significantly enhance the throughput of an imaging system. However, it should be noted that even remote focusing techniques involve moving parts, such as mirrors or lenses, within the optical path, and they can exhibit residual wavefront aberrations that limit their focusing performance across the entire lateral FOV^24^. This added hardware complexity and performance limitations can introduce challenges in the system design and maintenance, potentially offsetting some of the operational benefits. Transitioning from a mechanical to a completely computational PSF refinement (Fig. 2b) can be enabled using neural network-based refocusing approaches.

One of the most important metrics for adequate image refocusing and deblurring that has seen successful compromises and improvements through deep learning is the PSF, which characterizes the spatial extent of a point source in the acquired image, and its enhancement is pivotal for achieving sharper, more detailed images^25^. An out-of-focus or blurry image thus corresponds to a situation where the PSF is in some way enlarged and distorted. Deep learning has already demonstrated its ability to enhance the PSF^9,26–28^, improving high-resolution imaging. In the specific context of neural network compensation, numerous studies have already made significant strides^8–11,26^. These works, harnessing the power of deep learning, have not only managed to accelerate or greatly facilitate the imaging process but have also expanded the capabilities of microscopy systems to capture finer details and provide crisper, higher-quality images. In this section, we discuss various leading methods of PSF engineering and refinement, leveraging AI in fluorescence and brightfield microscopy, holography, and phase contrast microscopy.

One such technique is the single-shot autofocusing method termed Deep-R^8^. In this method, offline autofocusing^29^ is rapidly and blindly achieved for single-shot fluorescence and brightfield microscopy images acquired at arbitrary out-of-focus planes (Fig. 2c). Deep-R significantly accelerates the autofocusing process (about 15-fold faster) using an automated focused image inference, and all without any hardware modifications or the need for prior knowledge on defocus distances. A similar operation is accomplished with the network termed W-Net^9^, which comprises a cascaded neural network and a double helix PSF^30^, representing a noteworthy advancement in the context of virtual refocusing and consequential enhancement of the DOF. This deep learning-based offline autofocusing approach enhances the quality of image reconstruction while extending the DOF by ~20-fold. The W-Net model was developed as a sequence of two neural networks designed to enhance image quality through computational refocusing and reconstruction. The first part of the model works on adjusting a PSF-engineered input image to target specific planes within the sample volume. Following this initial calibration, the second network takes over, utilizing the virtually refocused images to conduct a comprehensive image reconstruction, as shown in Fig. 2d. This process is guided by a cross-modality transformation (wide-field to confocal)^31^, ultimately producing images that are comparable in quality to those obtained from confocal fluorescence microscopy. One way to view and combine these two approaches is: *a compromised PSF is exploited for purposes of speed and simplicity, and then rectified using AI*.

Deep learning-enabled refocusing has also been extensively demonstrated in holographic microscopy imaging^32–40^. Among such models is the enhanced Fourier Imager Network (eFIN) framework^11^. eFIN is a highly versatile solution for simultaneous hologram reconstruction, pixel super-resolution, and image autofocusing. eFIN enables sharper and higher-resolution imaging while maintaining image quality and is designed for both phase retrieval and holographic image enhancement on low-resolution raw holograms through its inference process. Building on the foundational Fourier Imager Network (FIN)^41^—a network that achieves better hologram reconstruction than convolutional neural networks (CNNs) by synergistically utilizing both the spatial features and the spatial frequency distribution of its inputs—eFIN showcases notable advancements in its model structure, particularly through the incorporation of a simplified U-Net within its Dynamic Spatial Fourier Transform (SPAF) module, which utilizes input-dependent kernels and better adapts to inputs with varying features. This innovative architecture enables eFIN to seamlessly combine pixel super-resolution with autofocusing functionalities within a singular framework. A distinctive feature of eFIN is its proficiency in autofocusing across an extensive axial range of ±350 μm, coupled with its remarkable ability to accurately estimate the axial positions of input holograms by leveraging physics-informed learning techniques^34^, thereby eliminating the reliance on actual axial distance measurements.

An altogether different kind of deep learning enhancement of the PSF is found in the rapid brightfield and phase contrast scanning method known as GANscan^10^. This powerful approach harnesses generative adversarial networks (GANs)^42^ to restore sharpness of images extracted from motion-blurred videos (Fig. 2e). In this case, the PSF is elongated horizontally and narrows the horizontal spatial frequencies. Adjusting for this defect, GANscan enables ultra-fast image acquisition through motion-blurred scanning. The resulting acquisition rate matches the leading-edge Time Delay Integration (TDI)^43^ technology’s performance, achieving 1.7–1.9 gigapixels within 100 s. Such a technique offers an efficient and cost-effective way to accomplish rapid digital pathology scanning using only basic optical microscopy hardware. Like the other methods in this section, GANscan serves to repair a damaged PSF, which then entails general imaging benefits.

Another approach^20^ demonstrates the same end goal of autofocusing with a phase contrast modality, but this time in a direct mechanical and signal processing fashion. Unlike the methods previously mentioned, this technique employs a neural network to predict the physical focal offset, which then prompts a change to the optical hardware. The PSF manipulation is, in this case, *mechanical* in nature, which precludes the injection of any false AI-generated image data. Using just one or a few off-axis light emitting diodes (LEDs), the method allows for a significant speedup in obtaining in-focus images, a critical factor for accurately capturing dynamic biological processes in real-time. The “fully connected Fourier neural network (FCFNN)” employed in this technique is designed to exploit the sharp features resulting from coherent illumination, allowing it to make accurate focus predictions from a single image (Fig. 2f). This concept aligns closely with some of the recent advancements in digital holography, where similar principles have been employed for rapid, post-experimental digital refocusing^44^.

It should be noted that many of these methods have successfully achieved real-time operation, demonstrating their practical viability. Techniques including Deep-R and W-Net operate at a speed of ~0.34 mm^2^/s on an Nvidia RTX 2080Ti graphics processing unit (GPU)^8,9^, making them suitable for dynamic and live imaging scenarios due to their rapid autofocusing capabilities. Additionally, eFIN can support real-time applications by integrating pixel super-resolution and autofocusing within a single framework at a speed of ~0.85 mm^2^/s on a consumer-grade GPU^11^. The fully connected Fourier neural network^20^ (FCFNN; Fig. 2f) with 2–3 orders of magnitude fewer parameters is also well-suited for real-time focus predictions and adjustments, optimizing hardware use to maintain focus during dynamic biological processes.

All these exemplary PSF optimizing instances collectively underscore the transformative potential of deep learning in microscopic image refocusing and deblurring, a critical factor in the pursuit of high-resolution imaging within the domain of biomedical imaging. By leveraging neural network compensation, these approaches speed up imaging and enhance microscopy systems, resulting in faster acquisition of sharper, high-definition images with better DOF.

## Reconstruction with less data

The quest for efficient image reconstruction in biophotonics often grapples with the challenge of data scarcity. In order to obtain high-quality images, especially for three-dimensional (3D) or quantitative systems, a large volume of measurements needs to be acquired, which entails longer imaging durations, more data, and in the case of live biological specimens, exacerbation of problems such as photoxicity^5^ and photobleaching^7^.

Purposely undersampling measurement data is thus a critical strategy in deep learning-enhanced biophotonics. This concept involves deliberately reducing the amount of data acquired during the imaging process, often entailing certain compromises in measurement metrics. However, the emerging trade-offs allow for various advantages, including increased imaging speed, reduced data acquisition requirements, and minimized photodamage to delicate samples. In this section, we explore how this practice has successfully been applied across a range of modalities, including Fourier ptychography, 3D fluorescence microscopy, optical coherence tomography (OCT), and digital holography, highlighting the diverse applications of this approach in enhancing biophotonic imaging with relatively sparse data.

**Fig. 3:**
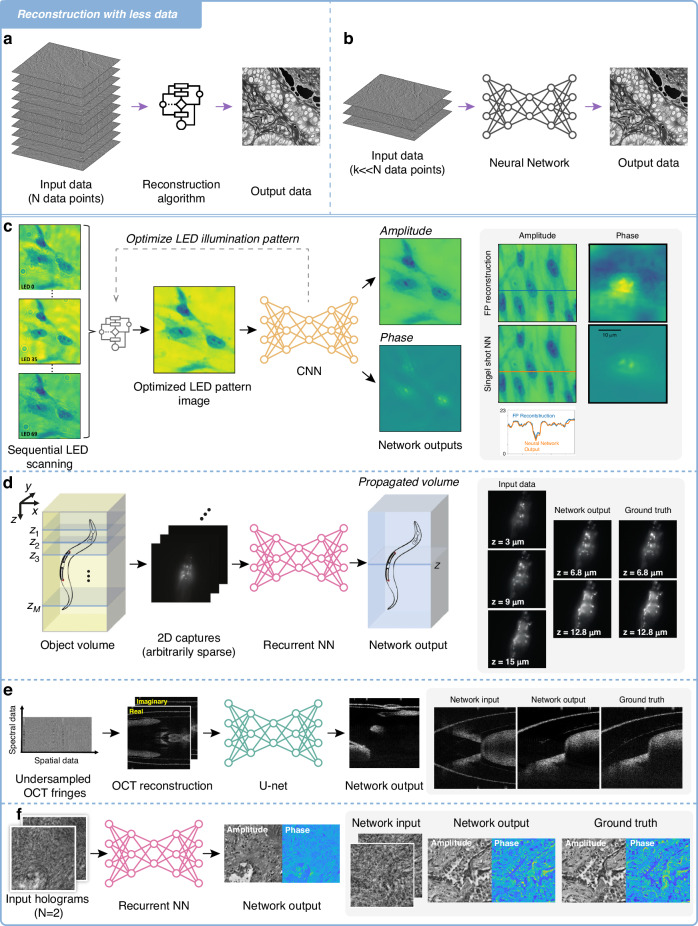
Reconstruction with less data. **a** Schematic representation of a typical reconstruction process. It consists of a dense set of input data and a standard algorithm. **b** With deep learning, a significant reduction from the traditional multi-layered stack is achieved for image reconstruction. This input is then fed into a neural network, which interprets and reconstructs the data. **c** Optimization of LED configuration using deep learning for Fourier ptychography with the resulting amplitude and phase components. An example from the evaluation dataset is provided for comparison, showcasing the phase component of the iterative Fourier ptychography reconstruction, which serves as the ground truth, alongside the output of the neural network, together with a cross-sectional analysis^15^ [figure adapted with permission from ref # 15 © Optical Society of America]**. d** The Recurrent-MZ volumetric imaging framework is illustrated through examples of 3D imaging of C. elegans, showcasing the initial input scans, the output processed by the network, and the established ground truths for comparison^12^. **e** The SS-OCT system acquires raw OCT fringes, from which the target image of the network is derived by directly reconstructing the original OCT fringes. By processing an undersampled image through a trained network model, an OCT image free of aliasing is produced, closely aligning with the ground truth. The provided example involves a 2× undersampled OCT image^14^. **f** Following a swift process of transfer learning, the RNN few shot hologram model demonstrates excellent generalization capabilities on test slides of new types of samples (lung tissue sections)

Typical image reconstruction processes require many input acquisitions that are then fed into an algorithm to generate a decent result (Fig. 3a). Employing a specially trained deep learning network, however, one may begin with a limited set of input data, a significant reduction from the traditional multi-layered stack, allowing for the minimization of initial data requirements without a meaningful loss in image quality (Fig. 3b). An example that applies this idea is the single-shot Fourier ptychographic microscopy method^15^, which introduces an important approach of strategically undersampling data and employing neural network compensation to nonetheless achieve high-resolution image reconstruction. Fourier ptychography is a computational imaging technique that enables high-resolution, wide-field imaging beyond the single-shot numerical aperture (NA) of the optical system employed. This method reconstructs a high-resolution image by stitching together information from a series of low-resolution images taken at different illumination angles^45^. Fourier ptychographic microscopy traditionally requires illuminating and capturing images from multiple LEDs in an array sequentially^46^. However, recent innovations have demonstrated that acquisition times can be significantly shortened through the use of multiplexed LED patterns^47^. Traditionally, the reconstruction of objects in Fourier ptychography, hindered by the loss of phase information in intensity images, relies on iterative algorithms that demand significant computational resources. Recent advancements have illustrated that deep learning can serve as an effective substitute for these iterative processes, streamlining the reconstruction method^48^. In this single-shot imaging methodology, the conventional ptychography LED illumination pattern is optimized using deep learning techniques, allowing for the acquisition of fewer images without compromising the SBP (Fig. 3c). Through the joint optimization of the LED illumination pattern and reconstruction network parameters, the deep learning model not only mitigates the impact of undersampling but also significantly reduces the acquisition time by a factor of e.g., 69^15^.

Another illustration of undersampling data can be shown with the deep learning-assisted volumetric fluorescent microscopy system that uses a model called Recurrent-MZ^12^. This recurrent neural network (RNN)^49^-based volumetric image inference framework utilizes 2D images sparsely captured by a standard wide-field fluorescence microscope at arbitrary axial positions within the sample volume. Through a recurrent CNN, Recurrent-MZ incorporates 2D fluorescence information from a few axial planes within the sample to digitally reconstruct the sample volume over an extended DOF, as shown in Fig. 3d. This approach significantly increases the imaging DOF of objective lenses and reduces the number of axial scans required to image the same sample volume, thereby advantageously undersampling data while maintaining imaging quality. These findings reveal that the Recurrent-MZ framework substantially enhances the DOF of a 63×/1.4 NA objective lens, achieving a remarkable 30-fold decrease in the necessary axial scans for imaging the same sample volume. This RNN-based framework has also been applied to undersampled image data in holographic microscopy^33^.

Similarly, in the context of OCT, the method known as Swept-Source OCT (SS-OCT)^14^ leverages a deep learning-based image reconstruction approach to generate OCT images using undersampled spectral data. OCT is a non-invasive interferometric imaging technique capable of delivering 3D insights into the optical scattering characteristics of biological matter^50^. This neural network-based SS-OCT approach eliminates spatial aliasing artifacts using less spectral data and without necessitating any hardware modifications to the optical setup. By training a deep neural network (DNN) on mouse embryo samples imaged by an SS-OCT system, researchers were able to blind-test the network’s ability to reconstruct images using 2–3 fold undersampled spectral data, as shown in Fig. 3e. The results showcase the network’s potential to increase imaging speed without compromising image resolution or SNR.

The concept of compensating specifically for less *training data* with DNNs has also been applied to the process of hologram reconstruction^51^. One such technique uses a few-shot transfer learning style for holographic image reconstruction^13^ that facilitates rapid generalization to new sample types using small datasets. Researchers pre-trained a convolutional RNN^33^ on a dataset with three different types of samples and ~2000 unique sample FOVs, which served as the backbone model. By transferring only specific convolutional blocks of the pre-trained model, they dramatically reduced (by ~90%) the number of trainable parameters while achieving equivalent generalization to new samples. An example of this on lung tissue image reconstruction is shown in Fig. 3f. Such an approach significantly accelerates convergence speed, reduces computation time, and improves generalization to new sample types, all while undersampling training data.

Many of these methods can potentially be used in real-time settings. For instance, the neural network-based SS-OCT approach highlights optimized inference time (as low as 0.59 ms on a cluster of 8 NVIDIA Tesla A100 GPUs) and is suitable for integration with existing OCT systems^14^. Techniques like single-shot Fourier ptychographic microscopy^15^ and Recurrent-MZ^12^ leverage deep learning to process sparse data rapidly, demonstrating at least one order of magnitude of acceleration compared to conventional methods. These methods inherently support faster data processing and reduced acquisition times, which are crucial for real-time imaging scenarios. As another example, the few-shot transfer learning approach for holographic image reconstruction^13^ facilitates rapid generalization to new sample types with small datasets, making it conducive to real-time imaging applications. With the rapid advancement of GPUs and neural processing units, efficient communication and control between the hardware and software may become the bottleneck for real-time applications. Future research could investigate extending these approaches to near real-time finetuning on individual samples, patients, and hardware.

The aforementioned methods highlight the strategic utilization of undersampled data in biophotonics and demonstrate how deep learning contributes to maximizing the advantages of this approach. Through deliberate compromise in data acquisition, these methodologies achieve enhanced imaging speed, reduced resource requirements, and minimized sample photodamage while maintaining or even improving imaging quality.

## Improving image quality and throughput

This section presents various deep learning-enabled approaches to enhance the *quality* of the biophotonic data and the *throughput* of the overall system using modest, cost-effective, or comprised equipment empowered by DNNs. In a similar vein to the previous methods discussed above, this approach leverages the power of neural networks to transform relatively suboptimal imaging data into high-quality representations, crucial for accurate biological analysis, all while forgoing some aspects of the optical hardware, including power, cost, and form-factor.

**Fig. 4:**
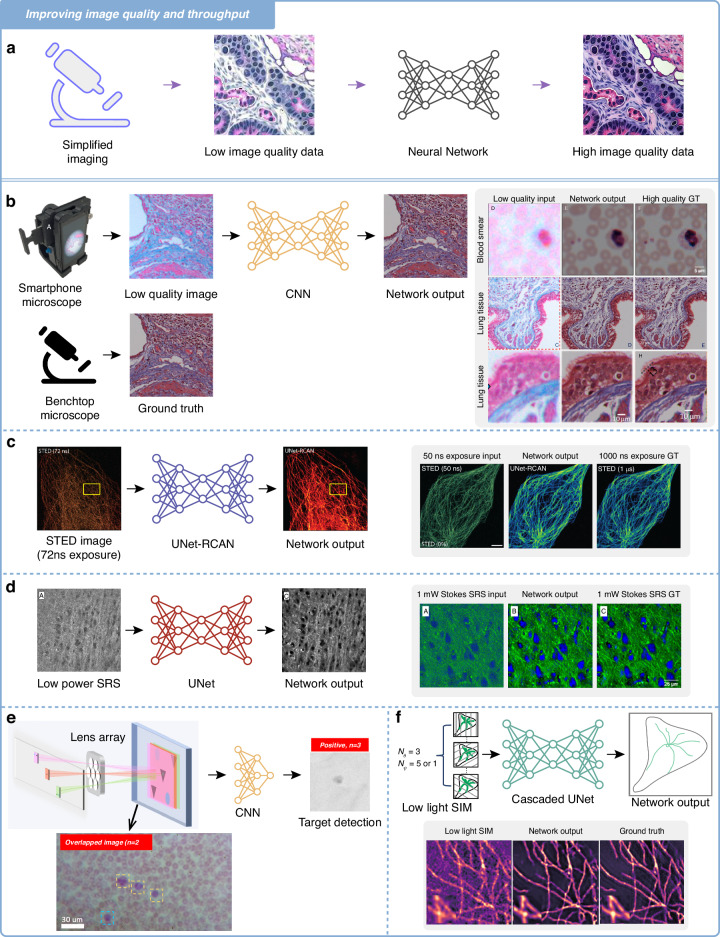
Improving image quality and throughput. **a** Schematic representation of a neural network-enabled pipeline for image quality improvement of data taken with simplified and/or inexpensive optics. **b** Deep learning enhanced mobile-phone microscopy with a CNN trained to denoise, color-correct, and extend the depth of field with examples of blood smears and lung tissue sections. **c** Low exposure STED SNR enhancement through UNet-RCAN. The example shown compares noisy images (exposure time of 50 ns), ground-truth images (exposure time of 1 μs), and images processed by UNet-RCAN on β-tubulin (STAR635P) in U2OS cells^21^ [figure adapted from ref #21, licensed under CC BY 4.0, http://creativecommons.org/licenses/by/4.0/]. **d** Reconstruction of low power (LP) SRS coronal mouse brain images and deep learning denoised versions, as well as two-color (lipids-green, proteins-blue) SRS images of a coronal mouse-brain slice with the ground truth as high power (HP) SRS images^19^ [figure adapted with permission from ref # 19 © Optical Society of America]. **e** The process of overlapped microscopy imaging involves illuminating various independent FOVs of samples using LEDs, followed by capturing these through a multi-lens array onto a shared sensor, resulting in an overlapped composite image. A CNN-based analysis framework is applied to detect and identify specific features within this composite image. This technique is exemplified by the model finding a target from an overlap of 3 images^17^ [figure adapted with permission from ref # 17 © Optical Society of America]. **f** Example of the low light SIM pipeline. For training the U-Net model, either fifteen (using three different illumination angles (*N*_θ_ = 3) and five phase patterns (*N*_ψ_ = 5)) with faint illumination or three SIM raw data images (a single phase pattern for fewer raw data acquisitions) are employed as input, while high SNR SIM reconstructions serve as the ground truth. This approach is shown with examples on microtubules^18^ [figure adapted from ref #18, licensed under CC BY 4.0, http://creativecommons.org/licenses/by/4.0/]

As depicted in Fig. 4a, traditional imaging systems using simplified devices invariably produce images of comparatively low quality in terms of SNR, spatial resolution, aberrations, and DOF. Rather than having to rely on hardware-intensive setups to achieve first-rate results, it is possible to compensate for these deficiencies using DNNs. Figure 4a showcases the application of a neural network to process images captured from a cost-effective optical microscope. Here, the network acts on a single low-quality image, eliminating the need for multiple captures and complex optical systems, and outputs an image that closely resembles one obtained from a high-end benchtop microscope.

With respect to image quality, maintaining a high SNR^52^ is of paramount importance. Achieving a higher SNR is critical for improving the sensitivity and accuracy of imaging techniques, especially in challenging conditions or when dealing with low light levels. Deep learning has emerged as a potent tool to augment SNR in such circumstances, leading to more reliable and informative imaging outcomes. A notable example of such an approach has been utilized on a type of mobile-phone microscopy^16^. While mobile phones have enabled cost-effective imaging technologies, their optical interfaces may introduce distortions/aberrations in imaging microscopic specimens, tampering with the SNR and image quality. Deep learning networks can correct these spatial and spectral aberrations, producing high-resolution, denoised, and color-corrected images that match the performance of benchtop microscopes with high-end, diffraction-limited objective lenses. This method standardizes optical images for clinical and biomedical applications, augmenting SNR and overall image quality (Fig. 4b).

A similar strategy was also presented in the paper titled “Deep learning enables fast, gentle stimulated emission depletion (STED) microscopy”^21^. In the realm of STED microscopy^53^, a technique that resolves features beyond the diffraction limit, the realization of super-resolution often comes at the cost of increased photobleaching and photodamage due to the necessity of high-intensity illumination. The use of deep learning in this paper aligns with the strategy of intentionally reducing pixel dwell time—thus sacrificing SNR and potentially image clarity—to improve the speed of imaging and reduce damage to biological samples, all while compensating for the sacrificed metrics using deep learning, as shown in Fig. 4c.

Another technique that is coupled with a similar power-saving process is stimulated Raman scattering microscopy (SRS)^54^, a label-free imaging modality that offers chemical contrast based on the vibrational properties of molecules within a sample. It operates on the principle of Raman scattering, where incident light interacts with the molecular vibrations of the sample, leading to a shift in the energy of the scattered light. Deep learning has now been integrated with this technique to deliver a promising solution to significantly improve the SNR of SRS images^19^. As depicted in Fig. 4d, a U-Net is trained to denoise SRS images of coronal mouse brain sections acquired with low SNR. The trained denoiser model also demonstrates external generalization to different imaging conditions, such as varying zoom and imaging depth, and augmenting SNR across various scenarios.

Another method involving overlapping FOVs^17^ effectively broadens the throughput of a microscopic imaging system by addressing the inherent limitation of the SBP in conventional microscopes. In traditional settings, the SBP requirements of a microscope hinders the capability to process wide areas quickly and efficiently without sacrificing spatial details. This overlapped imaging system^17^ includes a multi-lens array that circumvents the SBP bottleneck by capturing stacked images containing more information in a single snapshot, which can then be intricately processed and analyzed by an optimized machine learning model. This increases the throughput of the imaging process by a factor proportional to the number of FOVs that are integrated, allowing for a more efficient analysis of specimens, which is critical in biomedical research and disease diagnosis. This approach starts by lighting up different independent sample FOVs with LEDs. These are then imaged simultaneously through a multi-lens array onto a collective sensor, creating an overlapped composite image. To analyze this aggregate image, a CNN is designed to pinpoint and recognize distinct features or objects within it. Figure 4e shows an instance of this method, demonstrated through the model’s ability to locate a target blood cell from an image of 2 overlapped FOVs. This technique directly aligns with the strategy of using deep learning to compensate for and correct the compromised elements of biophotonic imaging setups, facilitating advancements in imaging capabilities. This approach not only augments the throughput of microscopic analysis but also exemplifies the major impact of deep learning in expanding the operational envelope of conventional biophotonic imaging methods. If the composite image were to be unraveled into its individual FOV constituents using DNNs, this capability could be used to significantly enhance various detection processes in different sample types, such as tissue sections.

Lastly, an imaging configuration making use of a low light source in structured illumination microscopy (SIM)^18^ showcases how deep learning improves SNR when imaging under extremely dim conditions. SIM, which works by illuminating the sample with patterned light, typically in the form of stripes or grids, and capturing multiple images as the pattern is shifted and rotated, thereby surpassing the optical diffraction limit, typically requires intense illumination and multiple acquisitions^55^. Deep learning facilitates the production of high-resolution, denoised images of faintly illuminated samples, as shown in Fig. 4f with microtubules. By enabling imaging with at least 100× fewer photons and 5× fewer raw data acquisitions (using fewer phase patterns in the illumination), this technique significantly boosts SNR, allowing for multi-color, live-cell super-resolution imaging with the added benefit of reduced photobleaching.

As highlighted through these examples, deep learning has significantly contributed to augmenting the SNR in biophotonics, enhancing the quality and reliability of imaging outcomes. These examples showcase how deep learning methods have effectively reduced noise, corrected distortions, and improved imaging volume, ultimately enhancing the SNR or the overall throughput of biophotonic imaging.

Furthermore, each of these methods presents a viable potential for real-time applications. For instance, deep learning-enabled mobile-phone microscopy could be integrated into portable and less power-intensive setups, allowing for immediate high-quality imaging and analysis in the field. The denoising of SRS images using a U-Net may be implemented in real-time^19^ to instantly improve image quality during live imaging of specimens, which is crucial for fast biological studies. Similarly, the overlapped microscopy technique^17^ coupled with CNNs can possibly process composite images to identify target features on-the-fly, enhancing the speed and efficiency of large-scale analyses. SIM^18^ can leverage deep learning to reconstruct high-resolution images from fewer acquisitions^56^, thus facilitating live-cell imaging without compromising on temporal resolution. These advancements illustrate the potential for deploying deep learning models in real-time settings, thereby transforming the practical applications of biophotonics imaging techniques and enabling more efficient and dynamic imaging workflows.

The techniques discussed in this section exemplify the effectiveness of applying deep learning to enhance image quality under suboptimal imaging conditions. These methods leverage the power of neural networks to compensate for hardware limitations, improve low image SNR, correct aberrations, and produce high-quality image data from cost-effective or compromised equipment. By doing so, they might facilitate the acquisition of reliable data while minimizing costs, increasing speed, reducing complexity, and minimizing photodamage.

## Discussion and future perspectives

The innovative integration of deep learning with biophotonic imaging represents a paradigm shift in bioimaging, offering a novel pathway to surpass traditional limitations and unlock new capabilities. This Review has shown how strategic compromises in measurement metrics, such as to the PSF, SNR, sampling density, and pixel resolution, can be effectively counterbalanced by designing and deploying specialized deep learning models. This approach not only substantially recovers lost information, but also enhances imaging parameters critical for advanced biophotonic applications, such as the resolution, FOV, DOF, and SBP. The successful applications of these strategies across various biophotonic methods underscore the transformative potential of deep learning in bioimaging, pushing the boundaries of what is achievable in terms of temporal resolution, imaging speed, accessibility, and cost-effectiveness.

An intriguing prospect with regards to this compromise–compensate scheme is the potential to combine different imaging defects strategically to further enhance or expedite imaging processes. For example, researchers can leverage a compromised PSF alongside low SNR to accelerate image acquisition. By deliberately introducing these imperfections, it is possible to optimize imaging speed without significant loss of critical information. The creative fusion of defects could offer exciting prospects for real-time imaging in applications where rapid results are imperative.

The synthesis of different deep learning methods also opens a realm of interesting possibilities. Consider integrating overlapped microscopy with a GANscan acquisition strategy. Overlapping multiple FOVs on a single image sensor can significantly increase detection throughput. When fused with GANscan’s already accelerated imaging capabilities, the result could revolutionize high-throughput imaging systems. There is, however, a legitimate concern that the adoption of these techniques in conjunction could face considerable pushback, for instance in scenarios where the precision and reliability of bioimaging are non-negotiable. The decision to leverage compromised imaging metrics for the sake of enhancing certain aspects of the imaging process, such as speed or FOV, necessitates a thorough and rigorous understanding of the trade-offs involved. It is essential to emphasize that the utility of these deep learning-enabled compromises is highly contingent upon the specific needs and constraints of the imaging task at hand.

Practically determining what constitutes a tolerable loss of initial information should be based on the specific biomedical application that the system is endeavoring to enhance. This is the ultimate measure of pertinence and means that the decisive metric should be the final accuracy of scientific classifications or pathology examinations, for example. The true measure of success in these scenarios is how well the imaging system can support such scientific findings or clinical decisions and contribute to, for example, accurate diagnoses, which are critical for patient outcomes. Therefore, future research should focus on validating imaging techniques based on their impact on specific biomedical targets of success, as opposed to mere technical quality metrics such as peak signal-to-noise-ratio (PSNR) or structural similarity index (SSIM).

Moreover, it should be noted that these methods frequently employ advanced models to predict missing information by learning from paired image datasets. Using deep learning-based techniques in this context poses a risk of obscuring rare phenomena and introducing biases that may counteract efficient measurement efforts. Deep learning models rely on existing data, where uncommon events are typically underrepresented. Consequently, these models may not adequately detect unexpected features, leading to generalization failure on shifted data distributions. Hence, it is vital to treat compromises or trade-offs gingerly to ensure that the integrity of scientific discoveries is not compromised and no regulations are overlooked. Uncertainty quantification (UQ) approaches of neural networks can be employed to mitigate potential risks caused by artifacts and hallucinations generated by these models. Most common UQ methods utilize Bayesian statistics^57–59^ and ensemble learning^60^, effectively providing quality control for neural networks’ outputs without access to the reference or ground truth data. Specifically for inverse imaging problems with known forward processes, Huang et al. demonstrated a cycle-consistency-based UQ, leveraging forward-backward cycles between physical forward models and corresponding trained neural networks^61^. Alternatively, generalization issues can be mitigated by performing transfer learning, parameter finetuning^13,62,63^, and physics-informed learning^34^.

In clinical diagnostics, where the accuracy and reliability of imaging data are critical for patient care, the acceptance of assorted optical flaws to expedite imaging processes might be especially risky. Conversely, in research settings where speed and scalability of imaging are more crucial, such compromises might be more readily welcomed. This underscores the importance of system-specific considerations in the application and wide adoption of these advanced imaging techniques. Therefore, it is imperative for researchers and practitioners to critically evaluate the potential benefits and limitations of deep learning-enhanced bioimaging methods within their specific contexts. The potential for hallucinations produced by various networks warrants careful examination. Since biomedical data often contain many subtle yet critical features that may be overlooked by inexperienced eyes, these models must be rigorously evaluated, especially when applied in separately trained systems. Moreover, using AI-generated content for training can amplify errors and reduce the accuracy of image reconstructions or transformations. It is essential to consider these factors diligently, along with appropriate labeling for medical professionals or experts dealing with these data to clearly indicate the generated results may be subject to contamination or hallucinations/artifacts. Understanding the precise requirements of their applications will allow the users to make more informed decisions about when and how to incorporate these innovative techniques, ensuring that the advancements in biophotonic imaging truly meet the nuanced demands of their work without unnecessarily sacrificing the quality or fidelity of the imaging data.

Finally, the path to Food and Drug Administration (FDA) approval with any of these systems remains a challenging milestone if the proposed systems are aimed to be used for diagnosing patients. Regulatory authorities often scrutinize compromised data, as it may raise concerns about unreliable medical results. However, the exponential growth in deep learning functionality and the vast wealth of data that is more and more at our disposal have the potential to address these concerns robustly. Deep learning algorithms, when rigorously validated and transparently documented, may prove, in time, their full reliability and safety. Furthermore, the generalization of AI models to learn from diverse datasets and adapt to different imaging conditions could mitigate the risks associated with compromised data. Demonstrating the significant benefits of deep learning in biophotonics will, however, demand utmost adherence to stringent regulatory frameworks and a comprehensive awareness of the potential pitfalls that need to be carefully examined, disclosed, and controlled, ideally in a self-supervised and autonomous manner, without access to ground truth.

As we stand on the cusp of this rapidly advancing field, it is clear that the future holds immense promise for myriad further innovations and breakthroughs.
